# Identification of a small molecule that simultaneously suppresses virulence and antibiotic resistance of *Pseudomonas aeruginosa*

**DOI:** 10.1038/srep19141

**Published:** 2016-01-11

**Authors:** Qiaoyun Guo, Yu Wei, Bin Xia, Yongxin Jin, Chang Liu, Xiaolei Pan, Jing Shi, Feng Zhu, Jinlong Li, Lei Qian, Xinqi Liu, Zhihui Cheng, Shouguang Jin, Jianping Lin, Weihui Wu

**Affiliations:** 1 State Key Laboratory of Medicinal Chemical Biology, Key Laboratory of Molecular Microbiology and Technology of the Ministry of Education, Department of Microbiology, College of Life Sciences, Nankai University, Tianjin 300071, China; 2 State Key Laboratory of Medicinal Chemical Biology and College of Pharmacy, Nankai University, Tianjin 300071, China; 3 State Key Laboratory of Medicinal Chemical Biology, College of Life Sciences, Nankai University, Tianjin, 300071, China; 4 Department of Molecular Genetics and Microbiology, College of Medicine, University of Florida, Gainesville, FL 32610, USA

## Abstract

The rising antibiotic resistance of bacteria imposes a severe threat on human health. Inhibition of bacterial virulence is an alternative approach to develop new antimicrobials. Molecules targeting antibiotic resistant enzymes have been used in combination with cognate antibiotics. It might be ideal that a molecule can simultaneously suppress virulence factors and antibiotic resistance. Here we combined genetic and computer-aided inhibitor screening to search for such molecules against the bacterial pathogen *Pseudomonas aeruginosa*. To identify target proteins that control both virulence and antibiotic resistance, we screened for mutants with defective cytotoxicity and biofilm formation from 93 transposon insertion mutants previously reported with increased antibiotic susceptibility. A *pyrD* mutant displayed defects in cytotoxicity, biofilm formation, quorum sensing and virulence in an acute mouse pneumonia model. Next, we employed a computer-aided screening to identify potential inhibitors of the PyrD protein, a dihydroorotate dehydrogenase (DHODase) involved in pyrimidine biosynthesis. One of the predicted inhibitors was able to suppress the enzymatic activity of PyrD as well as bacterial cytotoxicity, biofilm formation and antibiotic resistance. A single administration of the compound reduced the bacterial colonization in the acute mouse pneumonia model. Therefore, we have developed a strategy to identify novel treatment targets and antimicrobial molecules.

Bacterial resistance to currently available antibiotics is prevalent among many pathogenic bacterial species^1^. Meanwhile, the pipeline of new antibiotics development is running dry. An increased understanding of bacterial pathogenesis and antibiotic resistance has revealed many potential strategies to develop novel antimicrobials. In clinic, β-lactamase inhibitors have been used with β-lactam antibiotics to increase treatment efficacy^2^.

Molecules targeting bacterial virulence can disarm pathogens in the hosts, allowing host immune system and normal flora to prevent or eradicate the infection^3^. In the last decade, many antivirulence compounds have shown effectiveness in inhibiting infections of various pathogens in animal models, although large scale clinical trials are yet to be conducted^4^.

Computer-aided drug design is a widely used tool in pharmaceutical chemistry^5^. Once a target protein is determined, inhibitory molecules can be predicted based on the structure of the protein. This method has been successfully used to identify inhibitors of various enzymes, such as telomerase enzyme inhibitor for human cancer therapy and HIV-1 reverse transcriptase inhibitor for the treatment of HIV infection^5^,^6^,^7^. In this strategy, the identification of treatment target is critical for the successful development of novel drugs.

*Pseudomonas aeruginosa* is an opportunistic pathogen that causes morbidity and mortality in immunocompromised patients such as cystic fibrosis, AIDS, cancer patients and severe burn victims^8^. Multiple virulence factors are involved in the colonization of this bacterium. For example, *P. aeruginosa* directly injects bacterial effector molecules into host cell cytosol through a type III secretion system (T3SS), causing disruption of intracellular signaling or cell death^9^. Loss of the T3SS attenuates the bacterial virulence in mouse acute infection models^10^. During infection, *P. aeruginosa* secrets siderophores to acquire iron from host, which is essential for the bacterial growth^11^. The quorum sensing (QS) system regulates the expression of multiple virulence factors and plays important roles in the *P. aeruginosa* pathogenesis^12^.

Meanwhile, *P. aeruginosa* is notorious for its resistance to multiple antibiotics, which makes it one of the greatest therapeutic challenges. The resistant mechanisms include multidrug efflux pumps, low membrane permeability, inherent antibiotic modification enzymes, *etc*^13^,^14^. In addition, *P. aeruginosa* forms biofilm during chronic infection, which is highly resistant to antibiotics and immune clearance^15^,^16^.

Tremendous efforts have previously been put into the identification of inhibitors for either virulence factors or the mechanisms of antibiotic resistance^17^,^18^. However, molecules that can simultaneously suppress bacterial virulence and resistance would disarm the pathogen in host and make it vulnerable to drug and host clearance, thus might be superior to those with single target. The aim of the present work was to develop a strategy for the identification of molecules with dual functions, *i.e.* suppression of virulence as well as antibiotic resistance. We took advantage of the PA14 transposon (Tn) insertion mutant library^17^,^18^ to conduct *in vitro* screening and *in vivo* infection experiments, identifying PyrD as a promising antimicrobial target. One of the predicted PyrD inhibitors displayed inhibitory effect on both virulence and antibiotic resistance. Our results demonstrate that genes controlling both bacterial virulence and antibiotic tolerance can be identified through a comprehensive screening. Furthermore, utilizing a computer-aided inhibitor screening, molecules with dual inhibitory effects on virulence and resistance can be identified for the development of novel antimicrobials.

## Results

### Identification of genes that contribute to both antibiotic resistance and pathogenecity of *P. aeruginosa*

Our strategy to identify *P. aeruginosa* genes that are required for both antibiotic resistance and pathogenesis involves three steps: first, identification of mutants with increased antibiotic susceptibility; second, examine the cytotoxicity of the identified mutants; third, test biofilm formation abilities of those mutants with reduced antibiotic resistance and cytotoxicity.

**Figure 1:**
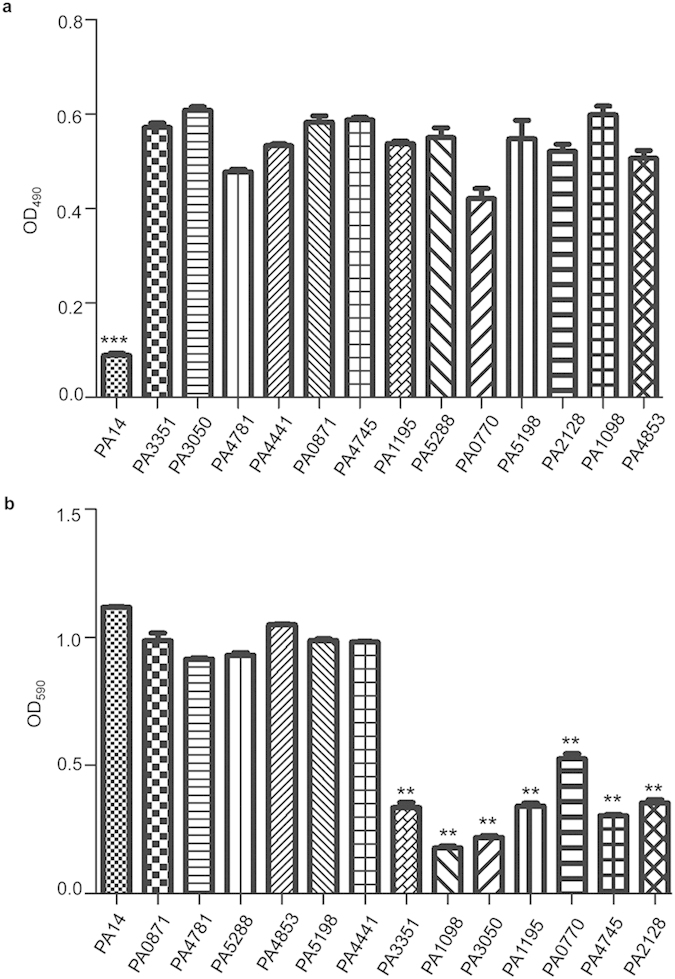
Screen for mutants with reduced cytotoxicity and biofilm formation. (**a**) Cytotoxicity of the PA14 Tn insertion mutants. HeLa cells were infected with the indicated strains at a MOI of 30. After 3.5 hours of infection, cells attached to the plate were measured with crystal violet staining. ***p < 0.001, compared to the other mutants by student’s t-test. (**b**) Biofilms formed by the Tn insertion mutants of PA14 were stained with crystal violet and quantified with optical density measurement. **p < 0.01, compared to the wild type strain by student’s t-test.

Previously, a comprehensive PA14 Tn insertion mutant library^19^ has been screened for mutants with altered antibiotic resistance^12^,^20^,^21^. Collectively, 93 mutants displayed increased susceptibility to various antibiotics (Supplementary Table S1). We then tested the bacterial cytotoxicity of those mutants by measuring their abilities to detach HeLa cells from culture plates. Out of the 93 mutants, 13 exhibited decreased cytotoxicity (Fig. 1a). Among those 13 mutated genes, PA1098, PA2128, PA3351, are related to motility, thus they may affect cytotoxicity through decreased attachment of the bacteria to the HeLa cells^22^. PA3050, PA0871, PA1195 and PA0770 encode enzyme for pyrimidine, nucleotide and energy metabolism, respectively. PA4781 encodes a cyclic di-GMP phosphodiesterase and PA5288 encodes a nitrogen regulatory protein which responds to cellular nitrogen levels. PA4745 is a transcription elongation factor and mutation in this gene results in impaired growth in LB and decreased virulence to *C. elegans*^23^.

Next, we tested biofilm formation by the 13 putative virulence-attenuated mutants. Compared to the wild type, seven mutants displayed reduction in biofilm formation by more than two folds (Fig. 1b).

Lastly, we performed MIC (minimum inhibitory concentration) assay to confirm the antibiotic susceptibility of the seven mutants that exhibited decreased cytotoxicity and biofilm formation. The antibiotics used in our tests included three aminoglycoside antibiotics, ciprofloxacin, tetracycline, erythromycin and carbenicillin (Table 1). Among the mutants, PA4745 and *pyrD* mutants showed increased susceptibility to at least four antibiotics. In comparison, mutation in the *pyrD* gene resulted in the most significant decrease in cytotoxicity, biofilm formation and antibiotic tolerance. Therefore, PyrD fulfills our criteria of potential therapeutic target.

**Figure 2:**
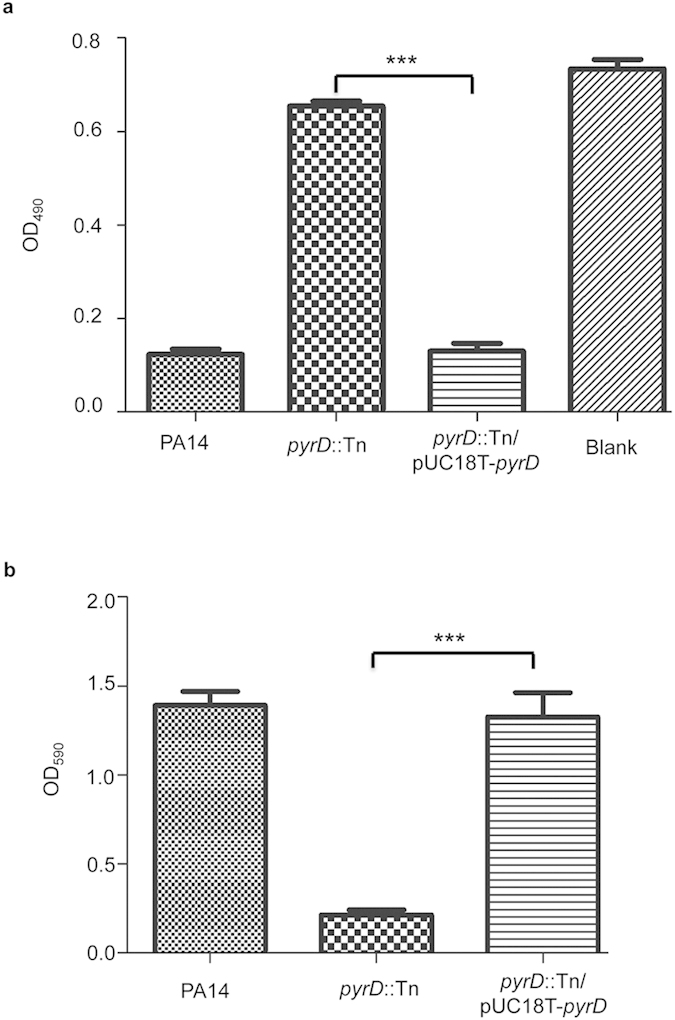
Role of PyrD in cytotoxicity and biofilm formation. (**a**) HeLa cells were infected with PA14, *pyrD*::Tn mutant and a complementation strain at a MOI of 30. After 3.5 hours of infection, cells attached to the plate were measured with crystal violet staining. ***p < 0.001, compared to the wild type or complementation strains by student’s t-test. (**b**) Biofilm formation by PA14, the *pyrD*::Tn mutant and the complementation strain. Results shown are means with standard deviations from three independent experiments. ***p < 0.001, compared to the wild type or complementation strains by student’s t-test.

In the *pyrD*::Tn mutant, the transposon was inserted 3 bp downstream of the start codon^19^. Complementation with a *pyrD* gene restored the bacterial cytotoxicity (Fig. 2a) and biofilm formation (Fig. 2b), confirming the role of PyrD in the observed phenotypes.

**Figure 3:**
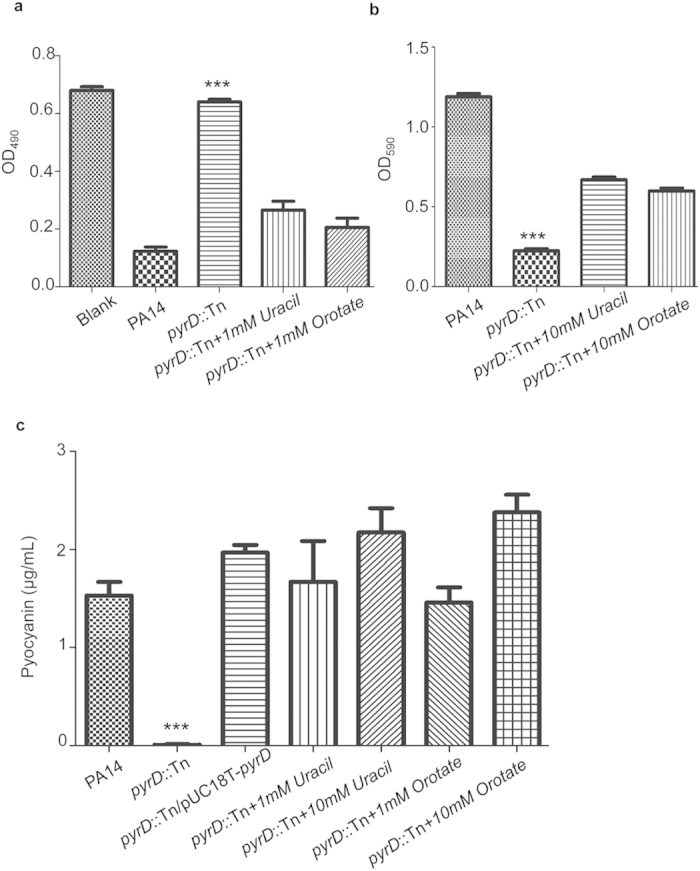
Orotate or uracil restores the cytotoxicity, biofilm formation and pyocyanin production of the *pyrD* mutant. (**a**) HeLa cells were infected with PA14, the *pyrD*::Tn mutant or the mutant supplemented with 1 mM orotate or uracil. Cells attached to the plate were measured with crystal violet staining. ***p < 0.001, compared to the wild type or mutants supplemented with orotate or uracil by student’s t-test. (**b**) PA14 was cultured in LB medium in the wells of a 96-well plate. The *pyrD*::Tn mutant was cultured in LB or with the supplementation of 10 mM uracil or orotate. 24 hours later, biofilm formation was measured with crystal violet staining. ***p < 0.001, compared to the wild type or mutants supplemented with orotate or uracil by student’s t-test. (**c**) PA14 and the *pyrD* complementation strain were inoculated in LB medium. The *pyrD*::Tn mutant was inoculated in LB or with the supplementation of 1 mM or 10 mM uracil or orotate. The bacteria were cultured at 37 °C. When the OD_600_ reached 2.0, the concentration of pyocyanin in each bacterial culture was determined. Each data represents the mean of at least three independent experiments. ***p < 0.001, compared to the wild type or complementation strains by student’s t-test.

As we grew the PA3050 (*pyrD*) mutant in LB, we noticed that the culture remained yellowish even at the stationary growth phase, whereas the culture of wild type PA14 turned green. This phenotype suggests a defect in the production of pyocyanin, a well known virulence factor^24^. Indeed, the pyocyanin production was severely reduced in the *pyrD* mutant, which was restored to wild type level when complemented by the *pyrD* gene (Fig. 3c).

### Supplementation of orotate or uracil partially restores the cytotoxicity, biofilm formation and pyocyanin production by the *pyrD* mutant

*pyrD* encodes DHODase which catalyzes the oxidation of dihydroorotate to orotate, the fourth step and the only redox reaction in the pyrimidine *de novo* biosynthesis pathway^25^. Therefore, mutation in *pyrD* can lead to deficiency in orotate and interruption of pyrimidine synthesis. Supplementation of 1 mM uracil or orotate during infection of HeLa cells restored the cytotoxicity of the *pyrD* mutant (Fig. 3a). Biofilm formation of the *pyrD* mutant was increased by the supplementation of 10 mM uracil or orotate (Fig. 3b). In addition, supplementation of uracil or orotate increased the pyocyanin production in a dose dependent manner (Fig. 3c). These results suggest that the observed phenotypes of the *pyrD* mutant are due to defective pyrimidine synthesis.

### *In vitro* expression of virulence related genes in the *pyrD* mutant

**Figure 4:**
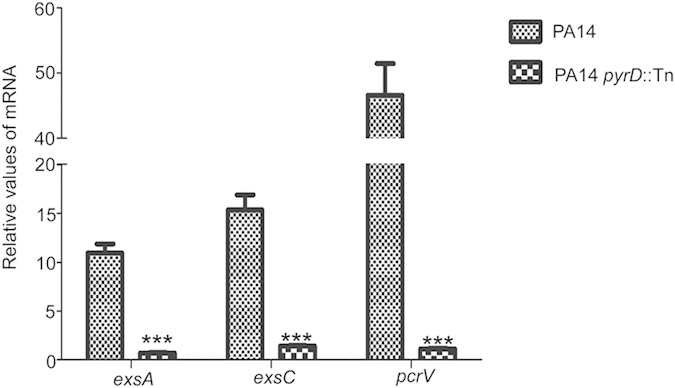
Expression of T3SS related genes under *in vitro* inducing condition. Wild type PA14 and the *pyrD*::Tn mutant were grown to an OD_600_ of 1.0 in LB containing 5 mM EGTA for 3 hours before RNA purification. The mRNA levels of *exsA, exsC*, and *pcrV* were determined by real-time PCR. ***p < 0.001, compared to the wild type strain by student’s t-test.

To further understand the role of *pyrD*, we examined the expression levels of genes related to cytotoxicity, pyocyanin production and biofilm formation. The cytotoxicity of *P. aeruginosa* is mainly mediated by T3SS^26^. The mRNA levels of T3SS genes, including *exsA, exsC* and *pcrV* in the *pyrD* mutant were significantly lower than those in wild type PA14 under T3SS inducing condition (Fig. 4). To further confirm the defective T3SS in the *pyrD* mutant, we knocked out the *pyrD* gene in another wild type strain PAK and examined the levels of ExoS (a T3SS effetor protein) by Western blot. Under T3SS inducing condition, the intracellular and secreted ExoS were reduced in the *pyrD* mutant (Supplementary Fig. S1). These results confirm the role of PyrD in T3SS. In addition, overexpression of *exsA*, which encodes a positive regulator of the T3SS regulon^27^, increased the expression of ExoS in the *pyrD* mutant, suggesting PyrD functions upstream of the ExsA (Supplementary Fig. S1).

The reduced pyocyanin production suggested a possible defect in quorum sensing system^24^. Accordingly, we tested the expression of quorum sensing genes at the OD_600_ of 2.0, when the quorum sensing system is activated. As shown in Table 2, the quorum sensing regulatory genes *lasI, lasR* and *rhlI, rhlR, mvfR* were down regulated in the *pyrD* mutant. And genes controlled by the quorum sensing system, including *lasB, chiC, rhlB, phzM*, were also down regulated. These results indicate a defective quorum sensing system in the *pyrD* mutant. Interestingly, expression of *pelA* and *pelB* genes, involved in the synthesis of exopolysaccharide, was reduced in the *pyrD* mutant (Table 2). The combination of reduced exopolysaccharide production and defective quorum sensing system may account for the defective biofilm formation by the *pyrD* mutant.

To explore the mechanism of increased susceptibility to antibiotics, expression levels of genes belonging to MexXY, MexAB-OprM and MexGHI-OpmD multidrug efflux systems were examined. Among these, we observed down regulation of *mexH* and *opmD* genes (Table 2), which are involved in the efflux of fluoroquinolone antibiotics and secretion of a PQS precursor to keep AHL homeostasis in *P. aeruginosa*^13^,^28^,^29^. Therefore, down regulation of the MexGHI-OpmD pump might contribute to the increased antibiotic susceptibility as well as defective quorum sensing system.

### PyrD is required for virulence in a mouse acute pneumonia model

**Figure 5:**
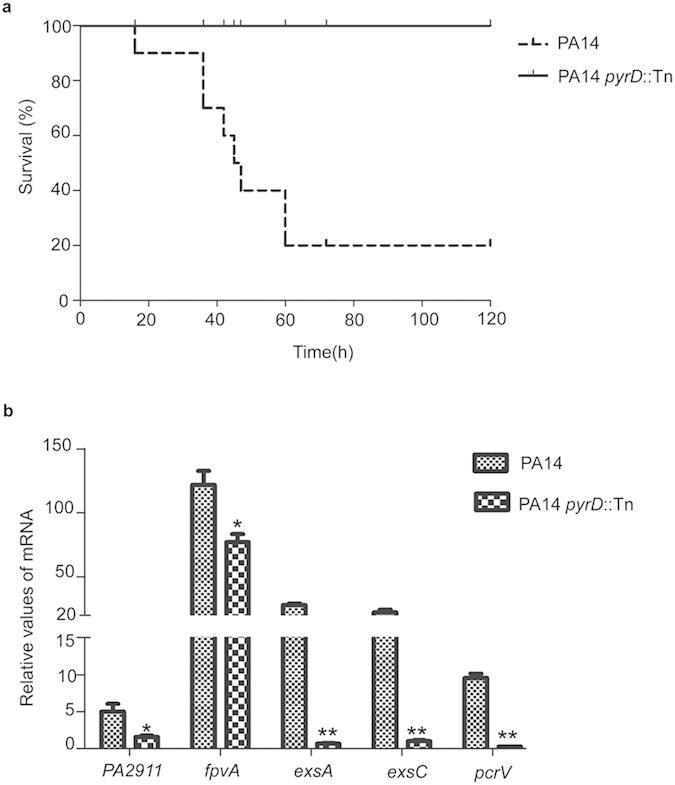
Role of PyrD in pathogenesis and virulence gene expression. (**a**) Survival of mice infected with either wild type PA14 or the *pyrD*::Tn mutant in an acute pneumonia model. *p* < 0.01 as calculated by Kaplan-Meier survival analysis with the Prism software (Graphpad software). (**b**) Expression of T3SS genes and iron acquisition genes during infection. Mice infected with wild-type PA14 or the *pyrD* mutant were sacrificed at 3 hours post infection. BALFs were collected and total RNA was isolated. The relative mRNA levels were determined by real time PCR. The 30S ribosomal protein coding gene *rpsL* was used as an internal control. *p < 0.05; **p < 0.01, by the Mann Whitney test.

The *in vitro* defective virulence factor expression suggests an attenuation of virulence in infection. To test for this, we compared the virulence of the *pyrD* mutant with wild type PA14 in a mouse acute pneumonia model. Sixty hours post infection with the same dose of bacterial cells, mice infected with the wild type PA14 resulted in 90% mortality while those infected with the *pyrD* mutant all survived (Fig. 5a).

To further understand the role of PyrD in infection, we examined the *in vivo* expression levels of virulence related genes. Bacteria in the bronchoalveolar lavage fluids (BALFs) were collected 3 hours post infection. Compared to the wild-type strain, the mRNA levels of T3SS genes *exsA, exsC* and *pcrV* were decreased at least 10-fold in the *pyrD* mutant. And genes related to iron acquisition, including *fpvA* and PA2911 were also significantly down regulated (Fig. 5b)^4^,^30^,^31^.

### Phenotypes of other pyrimidines and purines biosynthesis defective mutants

Our results described above suggest that defective pyrimidine biosynthesis is responsible for the reduced virulence and antibiotic resistance. If that is true, other genes in the pyrimidine synthesis pathway should also be required for the virulence and antibiotic resistance, and can serve as chemotherapeutic targets. From the PA14 transposon insertion mutant library, we picked strains with mutations in genes *carA, carB, pyrB, pyrC, pyrC2, pyrE, pyrF*, which are involved in the pyrimidine synthesis. Except for *pyrC* and *pyrC2* mutants, the other six mutants showed decreased cytotoxicity, biofilm formation and tolerance to several antibiotics (Table 3). Both the *pyrC* and *pyrC2* genes encode DHOase, which might compensate for the mutation of each other^32^. Therefore, we constructed a *pyrC* and *pyrC2* double knock-out mutant, designated as *pyrC’*. As expected, the *pyrC’* mutant displayed similar phenotypes as all other pyrimidine biosynthesis defective mutants (Table 3).

**Figure 6:**
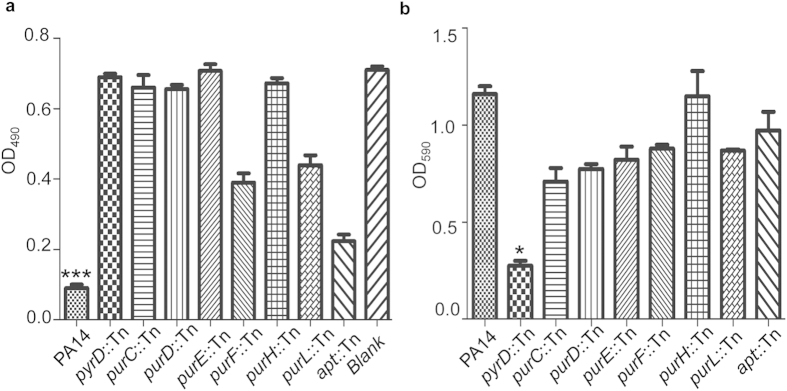
Phenotypes of purine biosynthesis defective mutants. (**a**) Cytotoxicity of wild type PA14 and the strains with mutation of *pyrD, purC, purD, purE, purF, purH, purL* or *apt*, as determined by the cell detachment assay. ***p < 0.001, compared to the other strains by student’s t-test. (**b**) Biofilm formation of the mutants was quantified by crystal violet straining. *p < 0.05, compared to the other mutants by student’s t-test.

Similar to the pyrimidine biosynthesis pathway, the purine biosynthesis is also involved in DNA and RNA metabolisms. Therefore, we tested the phenotypes of strains with mutations in the purine biosynthesis genes, including *purC, purD, purE, purF, purH, purl* and *apt*. Mutations in the *purC, purD, purE, purH* significantly reduced cytotoxicity (Fig. 6a), while mutations in the *purE, purL* and *apt* lead to partial reduction. Mutations in the *purC, purD, purE, purF* and *purL* modestly reduced biofilm formation (Fig. 6b). In comparison, the *pyrD* mutant exhibited similar levels of defects in cytotoxicity and biofilm formation as other pyrimidine biosynthesis gene mutants, but it shows the greatest reduction in antibiotic tolerance. Therefore, we chose PyrD as the chemotherapeutic target for further experimentations.

### Screen for small molecules targeting *P. aeruginosa* DHODase

**Figure 7:**
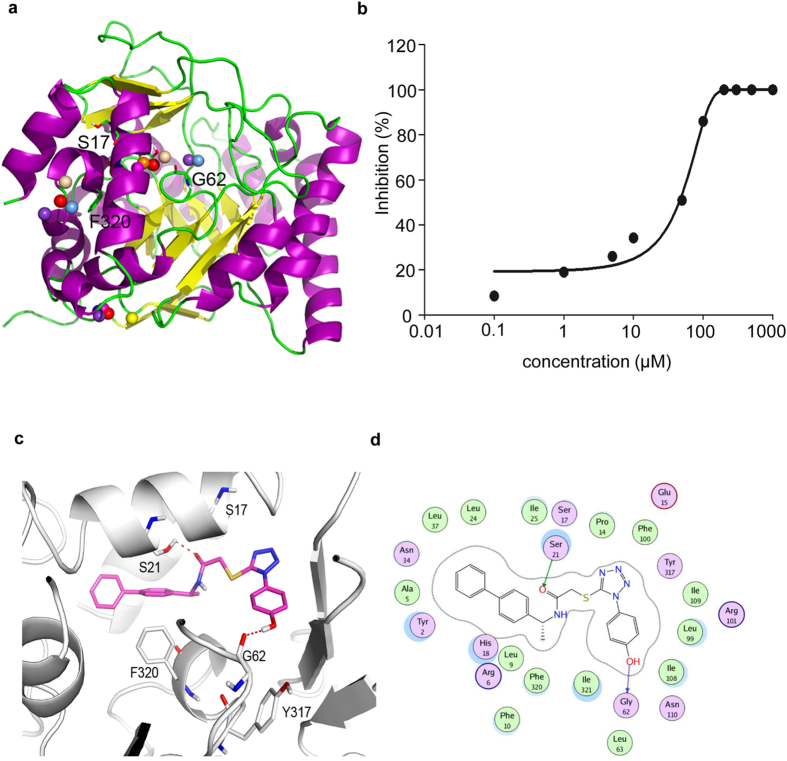
Model structure of the *P. aeruginosa* DHODase and concentration-dependent inhibition of recombinant *P. aeruginosa* DHODase by the compound 14. (**a**) Potential binding sites identified by the MetaPocket program. The spheres indicate volume of the binding site. (**b**) The line drawn through the data represents nonlinear least-squares fit to the equation: y = 1 × 100%/(1 + IC_50_/[*I*]), where [*I*] is the concentration of compound 14. IC_50_ is the concentration of the compound that leads to half-maximal inhibition of the enzyme. The least-squares analysis yielded an estimate of the IC_50_ for compound 14 inhibition of the recombinant DHODase of 300 nM. (**c**) Binding modes of compound 14 in the active site of *P. aeruginosa* DHODase. Dotted lines represent hydrogen-bonding interactions between ligand and DHODase. (**d**) 2D diagram of the interactions between ligand and active site.

The homology models of *P. aeruginosa* DHODase were constructed according to the structure of *E. coli* DHODase (PDB entry: 1F76, Resolution: 2.50 Å) (Fig. 7a). The quality of the constructed model was checked using PROCHECK (http://services.mbi.ucla.edu/SAVES/). It did not report any critical problems and all the constructed residues were found in the allowed region of the Ramachandran plot (Supplementary Fig. S2A).

Active site identification in the target protein is the starting point for virtual screening. MetaPocket 2.0 was employed to map possible binding pockets within the *P. aeruginosa* DHODase. MetaPocket identified three potential ligand binding sites. Based on further analysis of amino acid residues involved in the active site, the first pocket was chosen for the docking. The predicted active site is comprised of Tyr2, Ser17, Ser21, Gly62, Lys65, Arg101, Tyr317 and Phe320. To further identify the critical amino acid residues, each of the residues was mutated in the complementation plasmid (Supplementary Table S2) and transformed into the *pyrD* mutant. As shown in Fig. S2, PyrD with alteration in residues Ser17, Phe320 or Gly62 failed to restore the biofilm formation (Supplementary Fig. S2B) and pyocyanin production (Supplementary Fig. S2C), suggesting critical roles of these three residues.

The range of protein conformations arising from structural flexibility has a dramatic effect on a ligands binding pose^33^ and therefore greatly impacts drug discovery efforts. A 30 ns dynamics simulation was performed to get more reasonable conformations for the *P. aeruginosa* DHODase. One of the most important criteria to analyze the stability of the protein structure is to measure the root mean square deviations (RMSD). The deviations from the original starting structure have been measured over the course of simulation. RMSD increases rapidly at the beginning of the simulation, but stabilizes around 1.9 Å. Seven representative structures from the highly clustered conformations of MD simulation trajectory were selected for docking study.

A step-wise strategy for virtual screening was employed to identify allosteric inhibitors of *P. aeruginosa* DHODase. For this purpose, the Enamine database comprising of 200,000 compounds was docked in the active site of *P. aeruginosa* DHODase, utilizing the Glide’s High Throughput Virtual Screening (HTVS) scoring function to estimate protein−ligand binding affinities. This first screening resulted in the elimination of most of the molecules based on the application of GlideScore selection cut off of −8.0. The compounds were selected from the HTVS for further Glide SP docking study. The top 1000 compounds were selected on the basis of the docking score. In pursuit of selection of promising hits, visual inspection of compound binding mode was performed and the following criteria were considered: (1) protein ligand surface complementarity; (2) formation of hydrogen bond with residues in the active site, including Ser17, Gly62 and Tyr317. Finally, top scoring 17 compounds were purchased and subjected to the biological evaluation. The 2D structures, docking score and glide Emodel of hits selected for biological evaluation were listed in Supplementary Table S3^34^. And the physicochemical properties of the compounds were listed in Supplementary Table S4.

### Determination of DHODase inhibition

To test the inhibitory effects of the 17 selected molecules, we adopted a DCIP (2, 6-dichlorophenolindophenol)-based colorimetric assay. The DHODase catalyzes the oxidation of dihydrorotate (DHO) to orotate, and the colorimetric reagent DCIP is the final electron acceptor. DCIP reduction is stoichiometrically equivalent to oxidation of dihydroorotate^35^. We used a reaction system containing 200 μM Q_0_ and 150 μM DCIP as electron acceptors. The *K*_*m*_ and *V*_*max*_ values of DHO were determined as 60.8 μM and 12.9 μM/min, respectively (Supplementary Fig. S3A). Then we fixed the concentrations of DHO while varying the concentrations of the electron acceptor Q_0_. The *K*_*m*_ value for Q_0_ was determined as 25.6 μM and the *V*_*max*_ value was approximate 9.4 μM/min (Supplementary Fig. S3B). In combination, the appropriate enzyme reaction system was set as 200 μM Q_0_, 150 μM DCIP, and 1 mM DHO to ensure the maximum enzyme velocity.

For initial screening, we preincubated 0.3 μM enzyme with each of the 17 compounds (at a nominal concentration of 200 μM) for 30 minutes at 37 °C before addition of the substrates^36^. All the compounds except compound 14 showed weak or no inhibition at the concentration of 200 μM. As a control, the human DHODase inhibitor A771726 displayed no inhibitory effect (Supplementary Fig. S3C), which is consistent with a previous report^37^. In comparison, compound 14 exhibited the strongest inhibitory activity with an initial IC_50_ value of 41 μM (Fig. 7b). The possible interaction mode of compound 14 with the active site of DHODase was illustrated in Fig. 7c. The amide carbonyl and hydroxyl groups of compound 14 make a hydrogen bond with Ser21 and Gly62, respectively (Fig. 7c). Moreover, the diphenyl of compound 14 makes hydrophobic contacts with Leu37, Leu24, Ala5, Leu9, and Phe320 (Fig. 7d).

### Biological activities of compound 14

**Figure 8:**
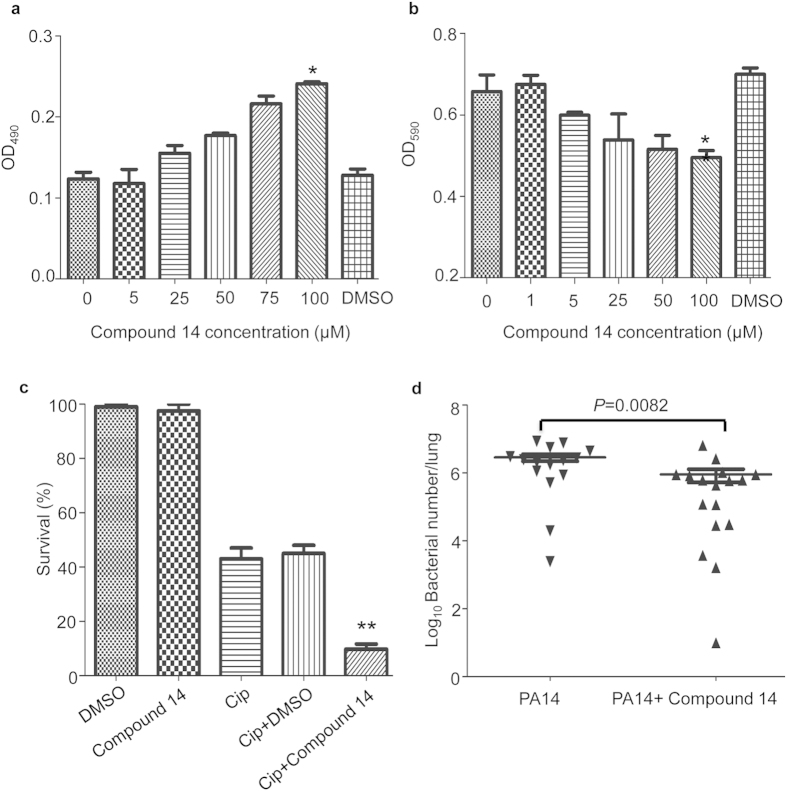
Biological activity of compound 14. (**a**) HeLa cells were infected with PA14 in DMEM without serum. Indicated concentrations of compound 14 were added to the medium. Cells attached to the plate were measured with crystal violet staining. The data shown represents three independent experiments. *p < 0.05, compared to the untreated sample by student’s t test. (**b**) Inhibition of early biofilm formation by compound 14. PA14 cells (5 × 10^8^ CFU) was incubated with indicated concentrations of compound 14 in LB in each well of a 96-well plate for 1 hour. The biofilm formation was measured with crystal staining. *p < 0.05, compared to the untreated sample by student’s t test. (**c**) Preformed biofilm (24-hour old) of PA14 was treated with 0.125 μg/ml ciprofloxacin alone or together with DMSO or 200 μM compound 14 for 3.5 hours. The biofilms were dissociated from the wells by gentle sonication and the bacteria were enumerated by plating. The bacterial survival rates were calculated based on live bacterial numbers in biofilms with or without treatment with ciprofloxacin (Cip). (**d**) 6–7 week old female BALB/c mice were intranasally inoculated with 7 × 10^6^ CFU of PA14 with or without compound 14 (400 μM). Eight hours post infection, mice were sacrificed. Lungs were dissected, homogenized and the bacterial loads were determined by serial dilution and plating. Bars represent medians and error bars represent SEMs. *p* = 0.0082, by Mann Whitney test.

Firstly, we evaluated the effect of compound 14 on the *P. aeruginosa* mediated cytotoxicity. Compound 14 itself displayed minimal cytotoxicity toward HeLa cell (CC_50_ > 500 μM) (Fig. S5). In the HeLa cell detachment assay, compound 14 and PA14 were added simultaneously. As shown in Fig. 8a, compound 14 reduced the detachment of HeLa cell in a dose-dependent manner.

To evaluate the effect of compound 14 on biofilm formation, we performed a rapid attachment assay as previously described^38^. As shown in Fig. 8b, compound 14 exhibited a moderate concentration-dependent inhibitory effect on the biofilm formation.

Next, we tested the effect of compound 14 on the antibiotic tolerance of biofilm. When the 24-hour old biofilm of PA14 was treated with DMSO or compound 14, no killing effect was observed (Fig. 8c). However, compound14 significantly enhanced the killing effect of ciprofloxacin (Fig. 8c).

Lastly, we evaluated the effect of compound 14 on the bacterial virulence in a mouse acute pneumonia model. Mice were infected intratracheally with 7 × 10^6^ CFU of PA14 mixed with DMSO or compound 14. Eight hours post infection, lungs were isolated and the bacteria were enumerated by serial dilution and plating. As shown in Fig. 8d, compound 14 reduced the mean bacterial load to approximately 20% of that infected with PA14 mixed with DMSO, suggesting an inhibitory effect of compound 14 on the bacterial virulence.

### Specificity of compound 14

**Figure 9:**
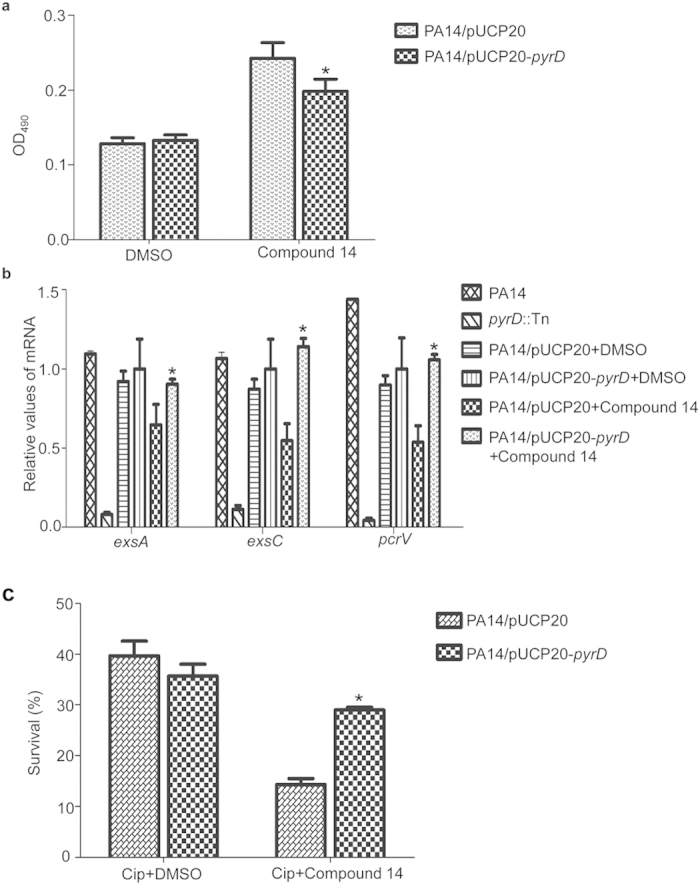
Overexpression of PyrD moderately reverses the biological activities of compound 14. (**a**) HeLa cells were infected with PA14 containing empty vector or *pyrD*-overexpressing plasmid in DMEM with 5% serum. 100 μM of compound 14 or DMSO were added to the medium. After 3.5 hours of infection, cells attached to the plate were measured with crystal violet staining. The data shown represents six independent experiments. *p < 0.05, compared to PA14 containing empty vector, by one-way ANOVA test. (**b**) 2.5 hours after infecting HeLa cells, the bacteria were collected from each well. Total RNA was purified and the mRNA levels of *exsA, exsC* and *pcrV* were determined with real time PCR. *p < 0.05, compared to PA14 containing empty vector treated with compound 14, by student’s t test. (**c**) PA14 containing empty vector or *pyrD* overexpressing plasmid were inoculated in wells of 96-well plate and incubated for 24 hour at 37 °C to form biofilm. Planktonic bacteria were removed. The biofilm was incubated in LB in the absence or presence of 0.125 μg/ml ciprofloxacin with DMSO or 200 μM compound 14 for 3.5 hours. The bacterial survival rates were calculated based on live bacterial numbers in biofilms with or without ciprofloxacin treatment. *p < 0.05, compared to PA14 containing empty vector, by one-way ANOVA test.

To test the target specificity of compound 14, we overexpressed PyrD in wild type PA14 and examined its effects on the biological activities of compound 14. Compared to the strain containing empty vector, the strain overexpressing PyrD displayed similar level of cytotoxicity on HeLa cells in the absence of compound 14. However, in the presence of compound 14, the PyrD overexpressing strain displayed higher level of cytotoxicity (Fig. 9a). To further confirm the effects of overexpression of PyrD on the T3SS, we tested the mRNA levels of T3SS genes during the cytotoxicity assay. In the *pyrD* mutant, the mRNA levels of T3SS genes *exsA, exsC* and *pcrV* were significantly lower than those in the wild type PA14. Compared to DMSO, compound 14 repressed the expression of those T3SS genes in PA14 containing empty vector. However, overexpression of PyrD diminished the inhibitory effect of compound 14 (Fig. 9b).

Next, we examined the biofilm susceptibility to ciprofloxacin. Overexpression of PyrD significantly increased the bacterial survival in the presence of compound 14 (Fig. 9c). These results suggest that the biological activity of compound 14 is likely through binding to block the PyrD function.

## Discussion

Targeting virulence and antibiotic resistance mechanisms is a compelling strategy for the development of novel antimicrobials^39^. Selection of the target is critical in this approach. The target must play essential roles in the bacterial pathogenesis or antibiotic resistance. The virulence factors include adhesins, toxins, specialized secretion systems and regulatory pathways of virulence genes, such as the quorum sensing system. Biofilm, multidrug efflux system and antibiotic modification enzymes are prevalent antibiotic resistant mechanisms^14^.

Two approaches have been widely used to identify small molecule inhibitors. One involves constructing a reporter system for the virulence or resistance determinant and conducting high throughput screening of small molecule libraries. However, it usually takes tremendous efforts to identify the targets of the inhibitory molecules. Screening overexpression mutants for resistant strains has been used to identify drug targets. However, Palmer *et al.* recently demonstrated that overexpression of drug targets can both increase or decrease resistance, depending on whether the drug only inhibits target activity or induces detrimental target catalyzed reactions^40^.

The other approach to identify small molecule inhibitors is a computer-aided inhibitor screening based on the structure of targeted proteins^6^,^41^. Many bacterial pathogens can cause acute and chronic infections, which might depend on different sets of virulence factors. For example, the T3SS plays critical roles in acute infections of *P. aeruginosa*, whereas the chronic infection is featured by biofilm formation^42^. Most acute infection associated virulence genes and those associated with chronic infection are reciprocally regulated in *P. aeruginosa*^43^. Therefore, it is likely that inhibitors of acute infection associated virulence factors can prevent colonization and even facilitate the elimination of pathogen at the early stage of infection, but they may not be able to affect biofilm formation. On the other hand, molecules targeting biofilm or antibiotic resistance mechanisms might not inhibit the acute infection associated virulence factors. A recent report demonstrated that *P. aeruginosa* bacterial cells dispersed from biofilm are highly virulent against cultured mammalian cells and *Caenorhabditis elegans* compared to planktonic cells, suggesting increased expression of acute infection associated virulence genes^44^. Thus, the dispersed bacteria might quickly colonize a new place, which is unlikely to be inhibited by an anti-biofilm drug. Therefore, molecules targeting multiple virulence factors and antibiotic resistance might be promising antimicrobials and can be used alone or in combination with established antibiotics to eradicate bacterial pathogen.

Here in this study, we firstly aimed at identification of proteins that are required for both virulence and antibiotic resistance. By utilizing the nonredundant library of PA14 transposon mutants^19^, we found that PyrD is required for multiple virulence factors, such as T3SS, quorum sensing, iron acquisition, biofilm formation as well as antibiotic resistance. However, the underlying mechanism remains elusive. We hypothesized that the defect in pyrimidine biosynthesis might lead to a stress response, which represses various regulatory pathways. It has been demonstrated that metabolic imbalance can lead to repression of T3SS in *P. aeruginosa*^45^. Since UTP is actively involved in polysaccharide metabolism, shortage of pyrimidine might affect the synthesis of extracellular polysaccharide, thus leading to defective biofilm formation. Surprisingly, mutants with defective purine synthesis displayed repressed T3SS, but normal biofilm formation. We suspect that either this pathway is not related to biofilm formation or the LB medium contains purine at the level high enough to sustain the biofilm formation.

Although all the genes in the pyrimidine biosynthesis pathway are required for T3SS, biofilm formation and antibiotic resistance, mutation in *pyrD* leads to the most profound defects in our experiment. The mechanism is unknown at present time.

Compound 14 modestly reduced the expression of T3SS genes and bacterial cytotoxicity. However, it significantly enhanced the killing efficacy of ciprofloxacin on biofilm. It might be due to different expression levels of PyrD in the planktonic and sessile modes of growth. Another possibility is that ciprofloxacin might enhance the entrance or biological effects of compound 14, or vice versa.

As *in vitro* experiments demonstrated inhibitory effect of compound 14 on PyrD, we overexpressed PyrD in wild type PA14 to test the target specificity of compound 14. Overexpression of PyrD partially counteracted the biological activities of compound 14, which might be due to insufficient expression level of PyrD or the presence of non-specific targets of compound 14. In addition, the IC_50_ of compound 14 on DHODase is in the micromolar range and the solubility of compound 14 is poor in various media. Further modification of the compound is needed to increase the solubility, cellular penetration, specificity and inhibitory efficacy.

The sequence alignment revealed particular sequence divergence in the N-terminal domains between bacterial and human DHODases (Supplementary Fig. S4), which might lead to differences in the active sites. Therefore, it is promising for the design of selective inhibitors that can specifically target the bacterial enzyme without influencing human counterpart.

Previously, it has been demonstrated that mutation in the genes of the pyrimidine synthetic pathway lead to defective biofilm formation in *P. aeruginosa* and *E. coli*^46^,^47^. Ueda *et al.* demonstrated that PyrF is required for quorum sensing, siderophore production, chemotaxis, type II secretion system and pili synthesis in *P. aeruginosa*^48^. In addition, the uracil analogue, 5-fluorouracil was able to repress the biofilm formation, and virulence gene in *P. aeruginosa* and *E. coli*^46^,^47^. In another study, 5-fluorocytosine was identified as an inhibitor of pyoverdine production^4^. Further studies revealed that 5-fluorocytosine inhibits the expression of *pchR* and *pvdS*, as well as virulence genes under the control of PvdS. In a mouse pulmonary infection model, 5-fluorocytosine protected the mice against infection by PAO1^4^.

Therefore, through different screening strategies, other groups and our laboratory identified that the pyrimidine synthetic pathway plays essential roles in the pathogenesis and antibiotic resistance of *P. aeruginosa, E. coli* and possibly other Gram negative pathogens. Molecules targeting the pyrimidine synthetic pathway might be promising antimicrobials.

## Methods

### Strains and plasmids

Bacteria and plasmids used in this work are listed in Supplementary Table S2. *P. aeruginosa* strains were routinely cultured in Luria-Bertani (LB) broth. Antibiotic concentrations used in this study were as follows: for *P. aeruginosa*, streptomycin at 200 μg/ml, tetracycline at 100 μg/ml, gentamicin at 10 μg/ml, carbenicillin at 150 μg/ml, neomycin at 100 μg/ml, and spectinomycin at 200 μg/ml; for *Escherichia coli*, ampicillin at 100 μg/ml, spectinomycin at 50 μg/ml, streptomycin at 25 μg/ml, gentamicin at 10 μg/ml, and tetracycline at 10 μg/ml.

### Cytotoxicity assay

Bacterial cytotoxicity was determined by a cell lifting assay as described before with minor modifications^48^. HeLa cells (1 × 10^5^) were seeded into each well of a 24-well plate and cultured in DMEM medium with 10% fetal calf serum at 37 °C with 5% CO_2_ for 24 hours. Overnight bacterial culture was subcultured into fresh LB to log phase before infection. Bacteria were washed once with PBS and resuspended in the DMEM medium. HeLa cells were infected with the bacteria at a multiplicity of infection (MOI) of 30. After 3.5 hours, the medium in each well was aspirated. Then, cells were washed twice with PBS and stained with 200 μl 0.025% crystal violet for 15 min at 37 °C. The staining solution was discarded, and the plates were washed twice with 1 ml phosphate-buffered saline. A 200 μl of 95% ethanol was then added into each well and incubated at room temperature for 30 min with gentle shaking. The ethanol solution with dissolved crystal violet dye was subjected to measurement for absorbance at the wavelength of 490 nm.

The CC_50_ of compound 14 for cultured Hela cells was determined as the concentration of the compound that inhibits 50% of MTT (3-(4, 5-dimethyl-2-thiazolyl)-2, 5-diphenyl-2-H-tetrazolium bromide) conversion to formazan^49^. Briefly, 8 × 10^3^ HeLa cells were seeded in each well of a 96-well plate. The cells were cultured for 24 hours at 37 °C in the presence or absence of various concentrations of compound 14. Then, MTT solution was added to each well, and the cultures were incubated for additional 4 hours. The supernatant was discarded and 150 μL DMSO was added to each well and the absorbance was recorded at 490 nm by a 96-well plate reader^50^. Values were determined from six repeats with concentrations of compound 14 ranging from 1000 to 7.8 μM.

### Biofilm formation and rapid attachment

Biofilm formation was determined using 96-well microtiter plates and was measured spectrophotometrically after staining with crystal violet as described by O’ Toole and Kolter^51^. Briefly, overnight bacterial cultures were diluted to an OD_600_ of 0.025 in LB and incubated in each well at 37 °C for 24 hours. For the quantification of biofilm formation, each well was washed twice with water and stained with 0.5% crystal violet, followed by two washes with water. Then 200 μl ethanol was added to each well. After 10 minutes of incubation at room temperature, optical density (OD) of each sample was measured at a wavelength of 590 nm.

Rapid attachment of bacterial to a solid surface was analyzed as previously described^38^,^52^. Briefly, overnight cultures were diluted into fresh MinA medium^53^ to an initial OD_600_ of 0.5 and mixed with various concentrations of the identified compounds or DMSO. Then 150 μl bacterial culture was added to each well of a 96-well polystyrene microtiter plate. Cells were allowed to adhere for 1 hour at 37 °C prior to staining with crystal violet.

### Determination of antibiotic susceptibility

MIC measurements for all bacterial strains were performed in liquid LB using a 2-fold serial dilution of antibiotics as previously described^54^. After incubation at 37 °C for 18 to 24 hours, the turbidity of each sample was determined by visual inspection, and the MIC was defined as the lowest concentration of antibiotic resulting in an optically clear bacterial culture. The test was repeated three times. The following antibiotics were tested: streptomycin, tobramycin, neomycin, erythromycin, carbenicillin, tetracycline, ciprofloxacin and meropenem.

For the biofilm killing experiment, biofilm was grown in each well of a 96-well plate for two days. Planktonic bacteria in each well were discarded and replaced with fresh LB. Then, the biofilm was treated with 0.125 μg/ml ciprofloxacin combined with various concentrations of the compounds or DMSO for 3.5 hours. Bacteria inside the biofilm was detached by sonication at a frequency of 40 kHz with a power output of 300 W for 5 min. Viable bacteria were enumerated by serial dilution and plating.

### Measurement of pyocyanin concentration

The pyocyanin quantification is based on the absorbance of pyocyanin at 520 nm in an acid solution^55^. Briefly, 1 ml of the bacterial culture was subject to centrifugation. The supernatant was collected and extracted with 600 μl chloroform. Then, the chloroform was transferred into a clean tube, and mixed with 0.5 ml 0.2 M HCl with gentle shaking to bring the pyocyanin to the pink aqueous phase. The absorbance of the aqueous phase was measured at a wave length of 520 nm and the concentration of the pyocyanin was determined by multiplying the optical density by 17.07^56^.

### Murine acute pneumonia model

All animal experiments complied with Chinese national and Nankai University guidelines regarding the use of animals in research. The protocol was approved by the Institutional Animal Care and Use Committee of the College of Life Sciences of Nankai University (Permit number: NK-04-2012). All the procedures were performed under anesthesia, and every effort was made to minimize suffering. The acute lung infection model was performed as previously described with minor modifications^57^. Briefly, wild type PA14 and the *pyrD* mutant were grown to an OD_600_ of 1. The bacteria were adjusted to 1 × 10^9^ CFU/ml in PBS. Each female Balb/c mouse (6 to 8-week old) was anesthetized by intraperitoneal injection of 100 μL 7.5% chloral hydrate. 10 μL of bacterial suspension was intranasally inoculated into each nostril of the anesthetized mouse. The mice were monitored at least twice a day for 5 days.

### RNA extraction and RT-PCR

To examine bacterial RNA levels during infection, mice were sacrificed by inhalation of CO_2_ 3 hours post infection. Bronchoalveolar lavage fluid (BALF) was obtained by cannulation of the trachea followed by two instillations of 1 mL PBS containing 0.5 mM EDTA. Bacteria were collected by centrifugation and resuspended in 200 μL TRIzol reagent (Invitrogen). Total RNA was isolated and further purified using a RNA cleanup kit (Tiangen Biotech). For *in vitro* grown bacteria, overnight cultures of bacterial cells were diluted 50-fold into fresh LB medium and grown to an OD_600_ of 2.0. Total RNA was isolated with an RNeasy Mini kit (Tiangen Biotech). cDNA was synthesized with reverse transcriptase (Takara) and random primers from each RNA samples. The 30S ribosomal protein gene *rpsl* was used as an internal control. Quantitative real-time PCR was conducted using the CFX Connect Real-Time system (Bio-Rad).

### Cloning, expression and purification of recombinant *P. aeruginosa* DHODase

The *pyrD* open reading frame was amplified from the genomic DNA of PAO1 and cloned into the NcoI/XhoI sites of pET28a^58^. The resulting plasmid was transformed into *E. coli* BL21. For large scale protein preparation, cells were grown at 37 °C in LB to an OD_600_ of 0.8. Expression of the protein was induced by addition of 0.1 mM IPTG and the bacteria were grown overnight at 16 °C. Cells were harvested by centrifugation, washed once PBS, and lysed by high pressure homogenization using an AH-BASIC apparatus (ATS Engineering Inc, Canada) in a running buffer (500 mM NaCl, 50 mM Tris, 5 mM imidazole, 0.1 mM FMN, pH 7.9). The lysate was centrifuged at 12000 rpm for 30 min. The supernatant was mixed with Ni-NTA agarose (Qiagen) for 1 hour at room temperature and washed three times with washing buffer (500 mM NaCl, 50 mM Tris, 20 mM imidazole, 0.1 mM FMN, pH 7.9). The protein was eluted with an elution buffer (500 mM NaCl, 50 mM Tris, 300 mM imidazole, PH 7.9). Then the protein was concentrated with a Micro Ultrafiltration system (Merck, Millipore) and dissolved in 50 mM Tris-HCl, pH 7.9.

### Computer-aided inhibitor screening

Homology modeling of *P. aeruginosa* DHODase was performed using MODELLER 9v12^59^. *P. aeruginosa* DHODase sequence (Accession ID: KGB88094) was retrieved from Uniprot database. Using NCBI Protein BLAST against PDB database, we identified *E. coli* DHODase as the best template for homology modeling of *P. aeruginosa* DHODase, due to high sequence identity. Thus, the three dimensional crystal structure of *E. coli* DHODase (PDB ID: 1F76) was retrieved from RCSB PDB database for use as the template in EASY MODELLER 4.0 software^60^. The best model was selected for further refinement, which comprised of loop refinement and energy minimization using Amber14. The minimized model was then validated using Ramachandran plot, generated from PROCHECK at the SAVES version 4 server (http://services.mbi.ucla.edu/SAVES/; Structure Analysis and Verification Server). The active site of the *P. aeruginosa* DHODase was predicted using the metaPocket 2.0 server^61^. To explore the structural flexibility of *P. aeruginosa* DHODase, AMBER14^62^ was used for dynamics simulation of modeled 3D structure, employing the ff99 force field^63^,^64^. The MD trajectories were clustered based on average linkage method and representative structures were taken to be used in virtual screening with Glide 6.1^65^, utilizing the high-throughput virtual screening (HTVS) and standard precision (SP) scoring function to estimate protein-ligand binding affinities. The center of the Glide grid was defined at the centroid of the active site residues. Default settings were used for both the grid generation and the docking. The high scoring compounds were inspected visually, and the selected compounds were subjected to biological evaluation. The compounds were purchased from TopScience (www.tsbiochem.com). Stock solutions (200 mM) of the compounds were made by dissolving the compounds in DMSO and stored at −20 °C.

### Enzyme activity assay and inhibition studies

Initial screening of the compounds was performed by a DCIP-based colorimetric assay. The reaction buffer is composed of 0.1 M Tris, 0.1 mM EDTA, 200 μM Q_0_, 150 μM DCIP, and 1 mM DHO, pH 8.0. The reaction was initiated by the addition of the enzyme and the loss of DCIP absorbance at 600 nm was monitored. Initial velocities were determined as the slopes of the progress curves at 10 min^66^.

Dose-dependent inactivation assay were performed by preincubating the enzyme solution at a final concentration of 300 nM in 50 mM Tris-HCl, pH 7.9 as described previously^36^. A total of 17 compounds were used for initial screening. The compounds (at nominal concentration of 200 μM) were preincubated with the enzyme for 30 min, followed by addition of the substrates to initiate the enzymatic reaction. The assay was repeated at least three times.

## Additional Information

**How to cite this article**: Guo, Q. *et al.* Identification of a small molecule that simultaneously suppresses virulence and antibiotic resistance of Pseudomonas aeruginosa. *Sci. Rep*. **6**, 19141; doi: 10.1038/srep19141 (2016).

## Supplementary Material

Supplementary Information

## Figures and Tables

**Table 1:** Mutations of *P. aeruginosa* that increase the susceptibility to different antibiotics.

PAO1 Ortholog	Gene Name	Fold decrease in MIC value compared to that of wild type PA14						
		Sm	Tob	Neo	Er	Cb	Tc	Cip
PA3351	*flgM*	4						
PA3050	*pyrD*	4	4	4	2			2
PA5288	*glnK*							
PA4441								
PA4781								
PA4853	*fis*						2	
PA2128	*cupA1*	2				2		
PA1195								
PA1098	*fleS*	2						
PA4745	*nusA*	2		2			2	2
PA5198		2				2		
PA0871	*phhB*							
PA0770	*rnc*	4		2		2		

Sm, streptomycin; Tob, tobramycin; Neo, neomycin; Er, erythromycin; Cb, carbenicillin; Tc, tetracycline; Cip, ciprofloxacin.

**Table 2:** Gene expression in *pyrD* mutant compared with wild-type PA14.

Gene ID	Gene Name	Change (n-fold ± standard deviation) in PA14 *pyrD*::Tn	Gene product
Quorum sensing related genes			
PA1430	*lasR*	−2.43 ± 0.322	Transcriptional regulator LasR
PA1432	*lasI*	−1.768 ± 0.006	Autoinducer synthesis protein LasI
PA3477	*rhlR*	−4.6 ± 1.695	Transcriptional regulator RhlR
PA3476	*rhlI*	−1.22 ± 0.999	Autoinducer synthesis protein RhlI
PA1003	*mvfR*	−12.26 ± 1.99	Transcriptional regulators
PA0996	*pqsA*	−4.18 ± 0.690	Cofactor biosynthetic process
PA0997	*pqsB*	−8.57 ± 1.44	Secondary metabolite biosynthetic process
PA3724	*lasB*	−13.78 ± 6.239	Elastase LasB
PA2300	*chiC*	−4.36 ± 1.418	Chitinase
PA3478	*rhlB*	−2.91 ± 1.23	Rhamnosyltransferase chain B
PA4209	*phzM*	−3.92 ± 0.247	Phenazine biosynthesis protein PhzM
PA4217	*phzS*	−1.47 ± 0.33	Phenazine biosynthesis protein PhzS
Biofilm formation			
PA3063	*pelB*	−5.73 ± 0.153	Exopolysaccharides component
PA3064	*pelA*	−3.56 ± 0.623	Exopolysaccharides component
Multidrug efflux system			
PA4206	*mexH*	−5.6 ± 0.140	Probable RND efflux membrane fusion protein precursor
PA4208	*opmD*	−3.5 ± 0.116	Probable RND efflux transporter

**Table 3:** Phenotypes of the pyrimidine synthesis defective mutants.

Mutated gene	PAO1 Ortholog	OD_490_(Cytoxicity)	OD_590_(Biofilm formation)	MICs (μg/ml)				
				Tob	Sm	Neo	Er	Cip
Wild type		0.08 ± 0.008	0.80 ± 0.004	2.5	50	25	150	0.25
*carA*	PA4756	0.57 ± 0.017	0.19 ± 0.008	1.25	25	25	150	0.25
*carB*	PA4758	0.62 ± 0.025	0.20 ± 0.005	1.25	25	25	150	0.25
*pyrB*	PA0402	0.48 ± 0.008	0.19 ± 0.003	1.25	12.5	25	150	0.25
*pyrC*	PA3527	0.08 ± 0.011	0.21 ± 0.014	2.5	25	25	150	0.25
*pyrC2*	PA5541	0.09 ± 0.005	0.74 ± 0.062	2.5	50	25	150	0.25
*pyrC’*	PA0401	0.59 ± 0.008	0.123 ± 0.018	1.25	25	12.5	150	0.25
*pyrD*	PA3050	0.61 ± 0.013	0.231 ± 0.038	0.625	12.5	6.25	75	0.125
*pyrE*	PA5331	0.55 ± 0.036	0.186 ± 0.012	1.25	12.5	12.5	75	0.25
*pyrF*	PA2876	0.42 ± 0.037	0.15 ± 0.004	0.625	25	6.25	75	0.25

Tob, tobramycin; Sm, streptomycin; Neo, neomycin; Er, erythromycin; Cip, ciprofloxacin.
