# Supplementary material for: Identification of a small molecule that simultaneously suppresses virulence and antibiotic resistance of Pseudomonas aeruginosa
Source: Sci Rep. 2016 Jan 11;6:19141. doi: 10.1038/srep19141 (PMC4707474; doi:10.1038/srep19141)
Supplement: Supplementary Information [file srep19141-s1.pdf]

Supplementary Information

Identification of a small molecule that simultaneously suppresses virulence and antibiotic resistance of *Pseudomonas aeruginosa*

Qiaoyun Guo[1], Yu Wei[2], Bin Xia[1], Yongxin Jin[1], Chang Liu[1], Xiaolei Pan[1], Jing Shi[1], Feng Zhu[1], Jinlong Li[2], Lei Qian[3], Xinqi Liu[3], Zhihui Cheng[1], Shouguang Jin[1, 4*], Jianping Lin[1, 2*] and Weihui Wu[1*]

[1] State Key Laboratory of Medicinal Chemical Biology, Key Laboratory of Molecular Microbiology and Technology of the Ministry of Education, Department of Microbiology, College of Life Sciences, Nankai University, Tianjin 300071, China; [2] State Key Laboratory of Medicinal Chemical Biology and College of Pharmacy, Nankai University, Tianjin 300071, China; [3]State Key Laboratory of Medicinal Chemical Biology, College of Life Sciences, Nankai University, Tianjin, 300071, China; [4] Department of Molecular Genetics and Microbiology, College of Medicine, University of Florida, Gainesville, FL 32610, U.S.A.

Table S1. Changes in susceptibility of *P. aeruginosa* mutants showing increased susceptibility.

Gene ID	Gene name	Gene function	Fold increased susceptibility									Reference
			β-lactams					quinolones			tetracyclines	
			Sam	Mem	Ctx	Ipm	Caz	Lvx	Nal	Cip	Tc	
PA0011		Putative 2-OH-lauroyltransferase				2	2					[20]
PA0334		Putative MFS transporter								2		[21]
PA0336	*ygdP*	Dinucleoside polyphosphate hydrolase								2		[21]
PA0337	*ptsP*	Phosphoenolpyruvate-protein phosphotransferase								2		[21]
PA0338		Hypothetical protein								2		[21]
PA0401		Noncatalytic dihydroorotase-like protein		2			2					[20]
PA0402		*pyrB*; aspartate carbamoyltransferase					2					[20]
PA0420		*bioA*, adenosylmethionine-8-amino-7-oxononanoate aminotransferase				4	2					[20]
PA0425	*mexA*	RND multidrug efflux membrane fusion protein	2		2		2	2	2	2	4	[12,21]
PA0426	*mexB*	RND multidrug efflux transporter	4		4		2	2	2	2		[12,21]
PA0427	*oprM*	Major intrinsic multiple-antibiotic-resistance efflux outer membrane protein OprM		2						2		[20,21]
PA0503		Putative biotin synthesis protein BioC				2						[20]
PA0572					2			2				[12]

PA0595		*ostA*				2				[12]
PA0702		Hypothetical protein						2		[21]
PA0703		Probable major facilitator superfamily transporter						2		[21]
PA0764	*mucB*	*mucB*; negative regulator for alginate biosynthesis		2	2	2	2			[12,20]
PA0766	*mucD*	*mucD*; serine protease MucD precursor	2		2					[20]
PA0770	*rnc*	*rnc*; RNase III	2			2	2		2	[12,20]
PA0794				2		2	2			[12]
PA0871						2				[12]
PA0928				2		2	2			[12]
PA0966	*ruvA*	Holliday junction DNA helicase						2		[21]
PA0967		*ruvB*		2		2	2			[12]
PA1005						2	2			[12]
PA1098	*fleS*	Two-component sensor						2		[21]
PA1011		Putative lipoprotein				1.5				[20]
PA1167				2		2	2			[12]
PA1195		Putative dimethylarginine dimethylaminohydrolase				1.5				[20]
PA14_07490/07510		Intergenic region				2				[20]
PA14_59780		*rcsC*; two-component system kinase sensor				1.5				[20]
PA1316				2		2				[12]
PA1375	*pdxB*	Erythronate-4-phosphate dehydrogenase						2		[21]
PA1483	*cycH*	*cytochrome*				1.5				[20]

		c-type biogenesis protein									
PA1588	*sucC*	Succinyl coenzyme A synthetase beta subunit							2		[21]
PA1611		Putative sensor/response regulator hybrid							4		[21]
PA1667		Hypothetical protein							2		[21]
PA1777	*oprF*	Major porin and structural outer membrane porin	2		2		2	2	2	16	[12,21]
PA1800	*tig*	Trigger factor							2		[21]
PA1801	*clpP*	ATP-dependent Clp protease proteolytic subunit							2		[21]
PA1802	*clpX*	ATP-dependent Clp protease ATP-binding subunit							2		[21]
PA1803	*lon*	Lon protease							4		[21]
PA1848					2		2	2			[12]
PA2008	*fahA*				2		2				[12]
PA2128	*cupA1*	fimbrial subunit CupA1					1.5				[20]
PA2432		Putative transcriptional regulator							2		[21]
PA2549		Hypothetical protein							2		[21]
PA2615	*ftsK*	Cell division/stress response protein		2	4	2.5	2	2	8		[12,20,21]
PA2800								2		4	[12]
PA2963		Putative aminodeoxychorismate lyase				2					[20]
PA2970	*rpmF*	50S ribosomal protein L32					1.5				[20]
PA3011	*topA*				2		2			16	[12]
PA3050	*pyrD*	dihydroorotate		1.5		2					[20]

		dehydrogenase								
PA3110				2		2				[12]
PA3160	*wzz*			2		2			2	[12]
PA3233							2		4	[12]
PA3262	*fklB*	*fklB*; put. peptidyl-prolyl *cis-trans*-isomerase, FkbP type			2					[20]
PA3351	*flgM*			2		2				[12]
PA3433		Putative transcriptional regulator, LysR family			2.5	1.5				[20]
PA3516		Probable lyase						2		[21]
PA3517		Putative lyase						2		[21]
PA3649		Putative membrane-associated zinc metalloprotease			2	1.5				[20]
PA3647	*ompH*					2	2			[12]
PA3670				2		2	2			[12]
PA3738	*xerD*	Integrase/recombinase *xerD*						4		[21]
PA3800		Conserved hypothetical protein				3				[20]
PA3818		*suhB*; extragenic suppressor protein SuhB	2			1.5				[20]
PA3976	*thiE*			2		2	2			[12]
PA3978		Hypothetical protein			5					[20]
PA4005		Conserved hypothetical protein			8	2				[12,20]
PA4007		*proA*; probable -glutamyl phosphate reductase			2.5					[20]
PA4069		Putative dTDP-4-rhamnose reductase-related protein	2		2					[20]

PA4088		Putative aminotransferase				3				[20]
PA4269	*rpoC*	DNA-directed RNA polymerase b chain				3	2		2	[12,20]
PA4393		Putative permease			2					[20]
PA4441				2		2	2			[12]
PA4456							2		2	[12]
PA4459		Hypothetical protein				2	2	2		[12,21]
PA4667		Hypothetical protein						2		[21]
PA4685		Hypothetical protein						2		[21]
PA4727	*pcnB*			2		2	2		4	[12,20]
PA4745	*nusA*	*nusA*; N utilization substance protein A		2	2	2	2		2	[12,20]
PA4753		Putative RNA-binding protein	2			3	2			
PA4781		Putative two-component response regulator						2		[21]
PA4853	*fis*						2		2	[12]
PA5130		Putative rhodanese-like domain protein			2.5					[20]
PA5174		Putative β-ketoacyl synthase			4					[20]
PA5198				2		2	2			[12]
PA5253	*algP*	Alginate regulatory protein AlgP						2		[21]
PA5280	*sss*	Site-specific recombinase						4		[21]
PA5288	*glnK*	nitrogen regulatory protein PII-2		3	2	1.5				[12,20]
PA5345	*recG*	ATP-dependent						4		[21]

		DNA helicase RecG			
PA5366	*pstB*	Phosphate ABC transporter, ATP-binding protein	2	2	[20,21]
PA5375	*betT1*	Choline/carnitine /betaine transporter family protein		2	[21]

SAM, sulbactam; Mem, meropenem; Ctx, cyclophosphamide; Ipm, imipenem; Caz, cephalosporins; Lvx, levofloxacin; Nal, nalidixic; Cip, ciprofloxacin; Tc, tetracycline.

Table S2 Strains and plasmids used in this study

Strain	Characteristics	Reference
P. aeruginosa		
PA14	Wild-type	[19]
pyrD::Tn	Mutant with transposon MAR2xT7 inserted in *pyrD* gene	[19]
*carA::*Tn	Mutant with transposon MAR2xT7 inserted in *carA* gene	[19]
carB::Tn	Mutant with transposon MAR2xT7 inserted in *carB* gene	[19]
pyrB::Tn	Mutant with transposon MAR2xT7 inserted in *pyrB* gene	[19]
pyrC::Tn	Mutant with transposon MAR2xT7 inserted in *pyrC* gene	[19]
pyrC2::Tn	Mutant with transposon MAR2xT7 inserted in *pyrC2* gene	[19]
pyrC'::Tn	Mutant with transposon MAR2xT7 inserted in *pyrC'* gene	[19]
pyrE::Tn	Mutant with transposon MAR2xT7 inserted in *pyrE* gene	[19]
pyrF::Tn	Mutant with transposon MAR2xT7 inserted in *pyrF* gene	[19]
purC::Tn	Mutant with transposon MAR2xT7 inserted in *purC* gene	[19]
purD::Tn	Mutant with transposon MAR2xT7 inserted in *purD* gene	[19]
purE::Tn	Mutant with transposon MAR2xT7 inserted in *purE* gene	[19]
purF::Tn	Mutant with transposon MAR2xT7 inserted in *purF* gene	[19]
purH::Tn	Mutant with transposon MAR2xT7 inserted in *purH* gene	[19]
apt::Tn	Mutant with transposon MAR2xT7 inserted in *apt* gene	[19]
pyrD::Tn/ pUC18T-*pyrD*	*pyrD*::Tn with *pyrD* inserted on chromosome with mini-Tn7T insertion; Tc^r	This study
PAKΔ*pyrD*	PAK with *pyrD* deleted	This study
PAKΔ*pyrD*/Ptac-exsA	PAKΔ*pyrD* with insertion of a single copy of ExsA driven by tac promoter at attTn7 sites	This study
Y317G	*pyrD*::Tn with pyrD (Y317 residue replaced with G); Tc^r	This study
K65G	*pyrD*::Tn with pyrD (K65 residue replaced with G); Tc^r	This study
G62F	*pyrD*::Tn with pyrD (G62 residue replaced with F); Tc^r	This study
S21G	*pyrD*::Tn with pyrD (S21 residue replaced with G); Tc^r	This study
S17G	*pyrD*::Tn with pyrD (S17 residue replaced with G); Tc^r	This study
R101G	*pyrD*::Tn with pyrD (R101 residue replaced with G); Tc^r	This study
Y2G	*pyrD*::Tn with pyrD (Y2 residue replaced with G); Tc^r	This study
F320A	*pyrD*::Tn with pyrD (F320 residue replaced with A); Tc^r	This study
**Plasmids**		
pUC18T-mini-Tn7T-Tc	For gene insertion in chromosome; Tc^r	This study
pUC18T-*pyrD*	*pyrD* gene of PA14 on pUC18T-mini-Tn7T-Tc; Tc^r	This study
pET28a	fusion protein expression plasmid; Kan^r	Novagen
pET28a-*pyrD*	Recombinant DHODase expression plasmid; Kan^r	This study
pTNS3	Helper plasmid	[26]
pE707	*exsA* gene of PAK on pUC18T-Mini-Tn7T-Gm driven by a *tac* promoter; Gm^r	[50]

Table S3. Chemical structure, docking score and glide Emodel of the selected 17 hit compounds.

NO.	Compound name	Compound structure	Docking score	Glide Emodel*
1	T5535460	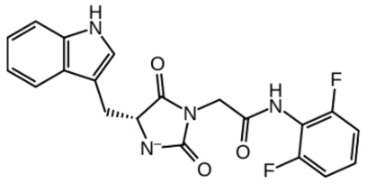	-10.48	-82.86
2	T6769616	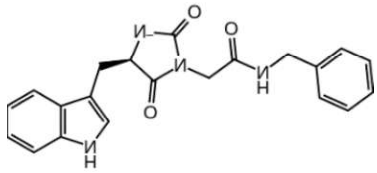	-8.81	-93.965
3	T5340649	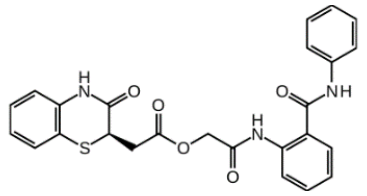	-10.95	-110.481
4	T5684873	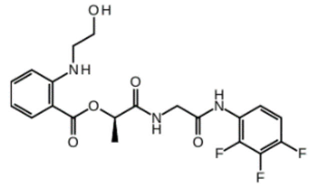	-10.63	-89.250
5	T6079767	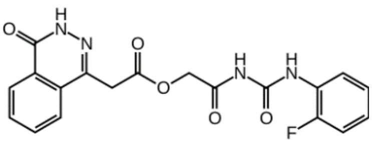	-9.23	-99.584
6	T05169024	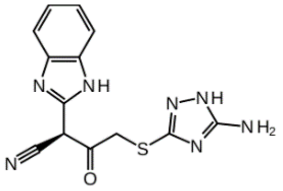	-9.71	-80.148
7	T5765833	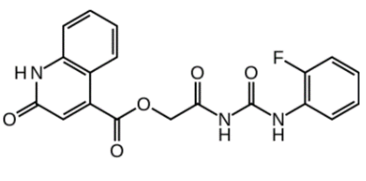	-8.24	-83.106
8	T5416992	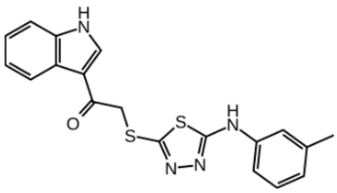	-9.24	-93.657

9	T5352049	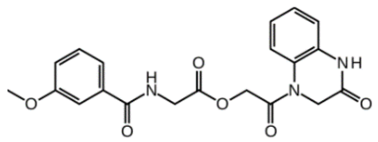	-10.05	-91.981
10	T05203411	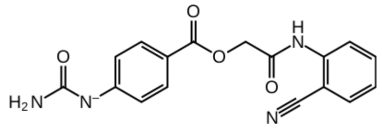	-8.58	-74.929
11	T5490496	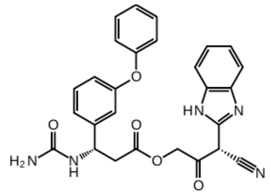	-10.87	-54.760
12	T5287811	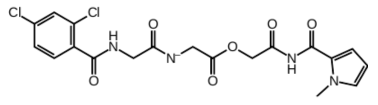	-9.49	-121.817
13	T5334297	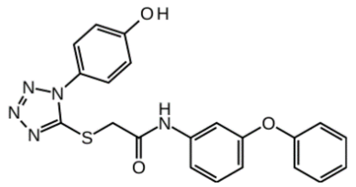	-9.05	-72.623
14	T5649060	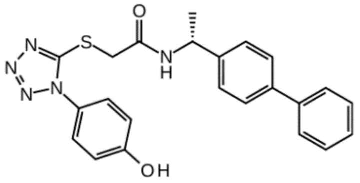	-10.64	-60.689
15	T6301657	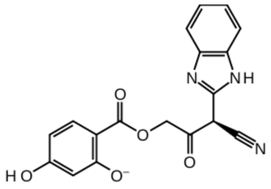	-7.68	-76.005
16	T5954234	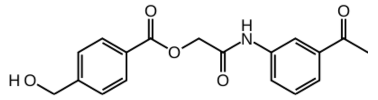	-9.08	-83.671

17	T5332630	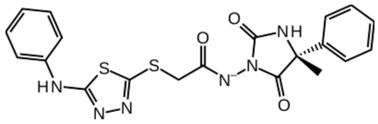	-7.76	-92.863

*Glide Emodel [36]: a model energy score that combines the energy grid score, the binding affinity predicted by GlideScore, and (for flexible docking) the internal strain energy for the model potential used to direct the conformation-search algorithm.

Table S4. Physicochemical properties of the compounds*

No.	MW[a]	QPlogP o/w[b]	QPPCaco[c]	QPlogHERG[d]	Percentage human oral absorption[e]	Rotatable bonds[f]	HBD[g]	HBA[h]	QPlogS[i]
1	398.4	3.2	322.1	-6.2	90.8	7	3	6	-5.3
2	376.4	2.7	218.1	-5.5	84.9	7	3	6	-5.0
3	475.5	3.9	210.3	-7.8	91.5	10	2	9	-6.6
4	439.4	2.9	122.7	-5.1	81.4	13	2	8	-5.1
5	398.4	2.0	30.9	-4.7	65.3	9	2	7	-4.6
6	313.3	0.6	22.3	-5.5	54.4	5	3	7	-4.3
7	383.3	1.6	50.9	-4.5	67.1	8	2	7	-3.9
8	380.5	4.7	379.9	-6.7	100	6	1	3.5	-6.6
9	397.4	1.8	107.3	-6.7	73.6	9	1	10	-4.7
10	338.3	0.04	10.1	-4.6	45.1	8	4	8	-3.5
11	497.5	3.4	19.4	-6.2	69.8	12	2	8	-6.3
12	469.3	2.9	65.4	-5.3	76.2	13	1.5	9	-5.6
13	419.5	3.6	152.1	-7.5	87.2	9	2	7	-6.0
14	431.5	3.6	111.4	-5.9	84.6	9	2	6	-5.6
15	351.3	1.8	29.3	-5.9	63.56	7	1	7	-4.9
16	327.3	1.5	104.4	-6.3	72.1	9	2	8	-4.2
17	454.5	3.7	148.9	-6.9	87.3	8	2	8	-6.4

[a] Molecular weight (acceptable range <500). [b] Predicted octanol/water partition coefficient log p (acceptable range from −2.0 to 6.5). [c] Predicted Caco-2 cell permeability in nm/s (acceptable range: <25 is poor and >500 is great). [d] Predicted value for blockage of HERG K+ channels (concern below −6.5). [e] Percentage of human oral absorption (<25% is poor and >80% is high). [f] Rotatable bonds (acceptable range <10). [g] Hydrogen bond donor count. [h] Hydrogen bond acceptor count. [I] aqueous solubility (acceptable range -6.0 to 0.5).

*, The physicochemical properties of the 17 hit compounds were calculated using the QikProp program.

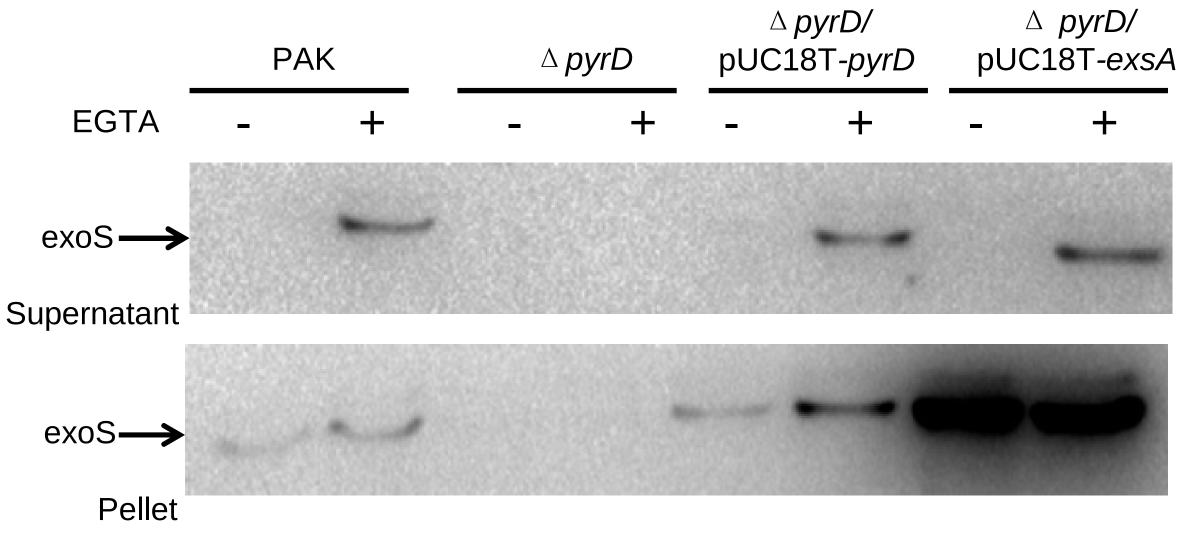

Figure S1 丨 Expression and secretion of ExoS in PAK, Δ*pyrD*, Δ*pyrD*/pUC18T-*pyrD* and Δ*pyrD*/P*tac*-ExsA mutant strains. Overnight bacterial cultures were diluted 50-fold in LB or 15-fold in LB plus 5 mM EGTA and grownfor 3h at 37℃. Proteins in the supernatants and pellets from equivalent bacterial cells were separated on SDS-PAGE, followed by immunoblotting with an anti-ExoS antibody. ExoS protein bands are indicated by arrows.

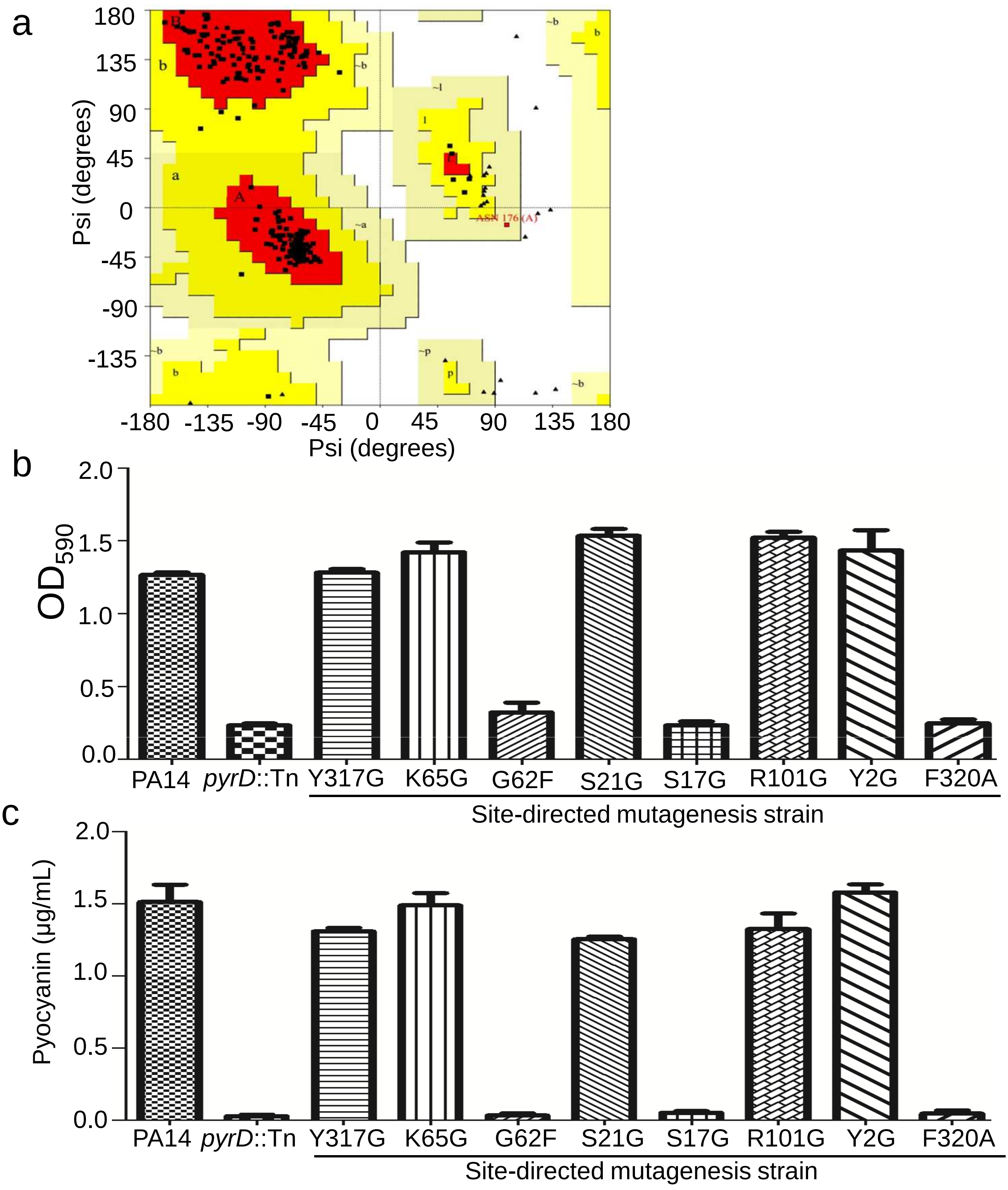

Figure S2 | The Ramachandran plot for the model of *P. aeruginosa* DHODase and phenotype of the DHODase point mutants. (**a**) The Ramachandran plot for the model of *P. aeruginosa* DHODase. (**b**) The *pyrD*::Tn mutant was complemented with a *pyrD* gene with indicated point mutations. Biofilm formation of the strains was determined by crystal violet staining. (**c**) The strains were inoculated in LB medium and cultured at 37℃. When the OD_{600} reached 2.0, the concentration of pyocyanin in each bacterial culture was determined. The data represents means of at least three independent experiments.

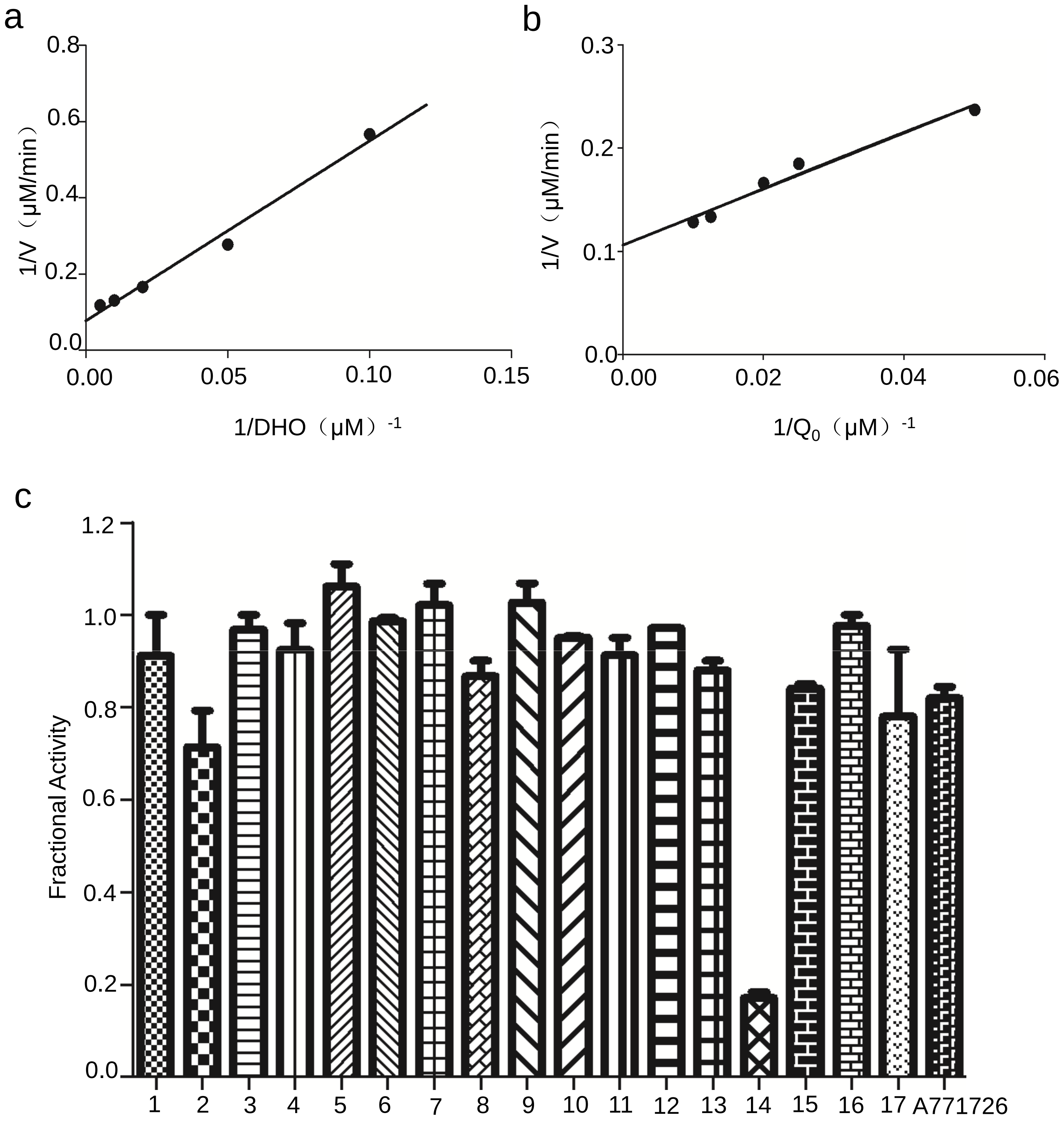

Figure S3 | Kinetic analysis of *P. aeruginosa* DHODase and inhibitor screening. Double-reciprocal plot of velocity data as a function of dihydroorotate concentration (**a**) or coenzyme Q_0 (**b**). (**c**) The observed enzyme activity with each compound (200 μM) was determined by preincubating the enzyme (300 nM) with the compound for 30 min before initiating the enzymatic reaction.

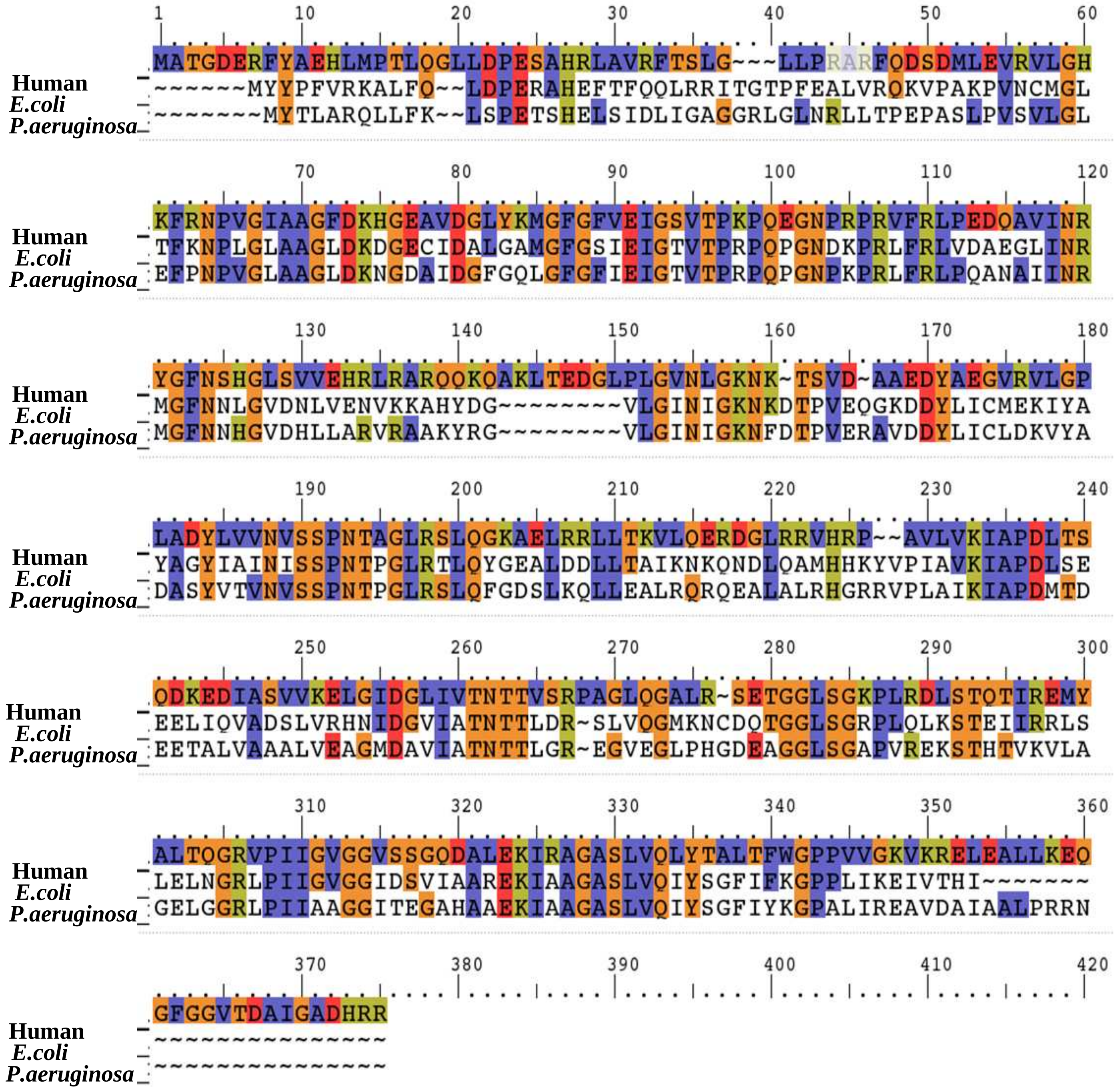

Figure S4 丨 Multiple sequence alignment of selected DHODase sequences. Residues are colored based on the degree of sequence conservation. The figure was prepared with the program Maestro 9.1.

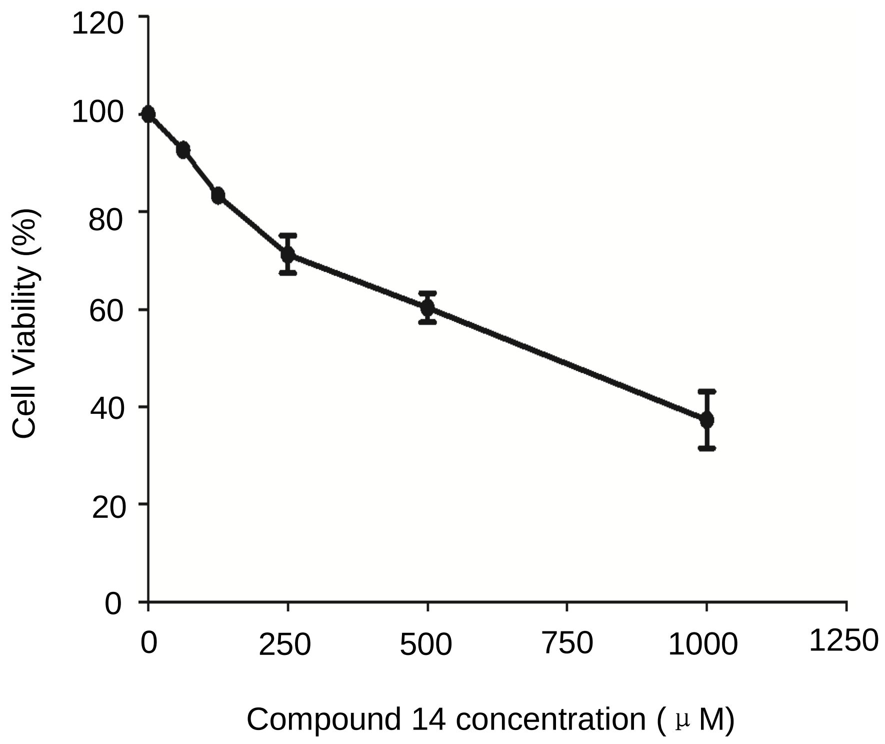

Figure S5 | Evaluation of the effects of compound 14 on HeLa cell growth. HeLa cells were cultured in DMEM medium in the presence of the indicated concentrations of compound 14 for 24 h. The growth of the treated cells was compared with that of untreated cells.
